# Combining a Cognitive Concurrent Task with a Motor or Motor-Cognitive Task: Which Is Better to Differentiate Levels of Affectation in Parkinson's Disease?

**DOI:** 10.1155/2020/2189084

**Published:** 2020-04-04

**Authors:** Arturo X. Pereiro, Bea Resúa, David Facal, José María Cancela-Carral

**Affiliations:** ^1^Universidade de Santiago de Compostela, Departamento de Psicoloxía Evolutiva e da Educación, Santiago, Spain; ^2^Universidade de Vigo, Department Specials Didactics, Faculty of Education and Sport Sciences, Vigo, Spain

## Abstract

**Introduction:**

Cognitive decline usually coexists with motor impairment in PD. Multitask settings provide appropriate measures to evaluate the complex interaction between motor and cognitive impairments. The main objective was to analyze which concurrent task, i. e., motor or hybrid motor-cognitive, in combination with a cognitive task better differentiates between PD patients with mild and moderate levels of disease.

**Methods:**

Thirty-seven individuals (19 male and 18 female) with idiopathic PD performed dual and triple tasks combining a cognitive task (phonemic fluency) with motor (pedaling) and/or cognitive-motor hybrid (tracking) tasks. Mild and moderate disability PD groups were specified considering the Hoehn and Yahr scale. Mixed ANOVA analyses for each of the concurrent task were carried out to test differences between the single and dual or triple condition performances comparing the low and high PD disability groups. Supplementary mixed ANCOVA analysis was performed considering the cognitive status as the covariate.

**Results:**

The only significant differences between disability PD groups were found for performances in the cognitive-motor hybrid (tracking) task, both in dual and triple conditions. Our results showed a better performance for the mild rather than for the moderate disability group in the single condition task and a significant decline of the mild disability group in the dual and triple condition when compared to the levels of those shown by the moderate disability group. The group-condition interaction remained significant when the cognitive status was statistically controlled.

**Conclusion:**

The hybrid of motor-cognitive task combining with a cognitive task (i. e., fluency) successfully differentiated between mild and moderate PD patients in the context of dual and triple multitask sets even when the cognitive status was statistically controlled. Our results highlight the importance of jointly measuring the complex interplay between motor and cognitive skills in PD.

## 1. Introduction

Parkinson's disease (PD) is mainly characterized by the progressive impairment of motor abilities [1]. However, cognitive decline usually coexists with motor impairment as a consequence of the neurodegenerative progression [2]. Despite the relationship between cognitive and motor impairments in PD [3], cognitive performance is usually measured regardless of the motor impairment or as with other nonmotor features of the illness, does not receive adequate attention [4].

The interaction between the motor and cognitive components seems to determine the functional capacity and should be considered simultaneously in the evaluation of the progression of PD [5]. Thus, voluntary movements in everyday life rarely are wholly automatic and therefore impose cognitive demands that could impair cognitive performance [6]. Conversely, because of loss of movement automaticity in PD, higher demands in attentional resources seem to be expected to maintain movement amplitude, rhythm, or posture [7, 8]. As some attentional resources must be allocated to the voluntary movement, performing a concurrent cognitive task may well interfere in the motor performance of PD patients [9].

The concurrent-task paradigm (multitask setting) constitutes an appropriate paradigm to evaluate the reciprocal influences between the motor and cognitive components because reciprocal costs can easily be estimated considering how the performance in both motor and cognitive tasks is hindered in concurrent condition regarding its performances in the single condition. The analysis of the relationships between cognitive and motor impairments in PD should be useful to assess the progression of symptoms, optimize the effectiveness of interventions, and finally, improve the patient's quality of life [10]. The concurrent-task paradigm provides a way of measuring the ability to share attentional resources in order to attend to the requirements of the two or more concurrent tasks (e.g., usually two, “dual,” or at most, three concurrent tasks, “triple”) devoted to the measurement of either cognitive, motoric, or both skills [11]. Since PD leads to a loss of automaticity in motor actions that compete for the available attentional resources, execution in the dual condition is expected to decline, particularly when the concurrent tasks impose high motor and cognitive demands. Despite the subsidiary motor and cognitive processes that are generally involved in most experimental tasks (e.g., perceptual processes and motor responses), the most demanding cognitive or motor component of a task corresponds to those that rely on voluntary processes, more resource-consuming, and those that can be considered core processes to achieve the main task-goal.

As far as we know, no study has been conducted to analyze the influence of the triple condition in patients with PD, but considering what has been reported for healthy older adults [12], a decline in performance compared to that observed in the dual condition could be expected also in PD patients.

Some studies carried out on healthy older adults have pointed out that the multitasking costs (i. e., performance worseness in the concurrent condition compared to the single condition) were higher in motor-cognitive than in motor-motor task sets [13–15]. Evidence from research in PD patients is not conclusive. Thus, although some studies found increased dual costs in motor-cognitive dual task sets [14, 16], some evidence pointed out similar dual task costs for motor-motor and motor-cognitive dual task sets [17]. Even when higher dual costs are associated with motor-cognitive dual task sets, it is not clear which of the concurrent tasks, cognitive or motor, might lead higher dual costs. In addition, the specific effects that a more hybrid motor-cognitive concurrent task could impose on the cognitive load when it is performed in a multitask setting should also be considered. Measurement of dual costs should be made carefully in order to prevent confounding the dual effect with differential demands associated to the disability level [18–20] or interference between processing mechanisms involved in both concurrent tasks [21]. Thus, multitask costs could be artificially increased when concurrent tasks involve similar processes for the input processing (e. g., visual and auditory), the selection of responses or the stimulus-response mapping according to the task response-criteria. In these conditions, the organization of the mental-set will be more difficult, and concurrent performance will be more prone to error and slowness [21].

Our aim was to elucidate which concurrent task (i. e., motor or hybrid motor-cognitive) in combination with a cognitive task is better to differentiate between PD patients with mild and moderate levels of disease [22]. Three tasks, namely, phonemic fluency (cognitive), pedaling (motor), and tracking (hybrid), were performed in single, dual (fluency-pedaling; fluency-tracking), and triple (fluency-pedaling-tracking) conditions. Single, dual, and triple performances were compared between mild and moderate disability PD groups, with cognitive status statistically controlled for. Among the concurrent cognitive tasks used in PD patients, verbal fluency tasks have been extensively studied [23, 24]. Unlike action fluency [25–28], phonemic fluency seems to be relatively unimpaired in PD [23, 24]. Regarding motor tasks, automated motor activities such as cycling or pedaling are good candidates for concurrent tasks since they provide purer motor measures and prevent cognitive interference in motor performance. Results have shown that these tasks seem to be largely preserved in PD patients [29, 30], and even improvements in dual costs have been reported for several cognitive measures in patients with PD [31, 32] and other motor concurrent tasks in healthy older adults [11], when this concurrent task is implemented in dual paradigms. The tracking test is a simple task that requires visuospatial and fine motor skills to quickly cross a series of circles arranged sequentially. This task was developed specifically to reliably measure dual-tasking ability in clinical practice [33] and unlikely to interfere with the main cognitive (i. e., lexical access) and motor (i. e., automated lower limb movement) processing mechanisms, respectively, involved in fluency and in pedaling.

In light of dual task taxonomy by McIsaac et al. [11], higher dual costs are expected for concurrent tasks showing higher complexity and novelty. Therefore, fluency-tracking should be more likely to show dual costs than fluency-pedaling and also be more likely to be observed in the Fluency and Tracking concurrent tasks because more complexity and novelty are implicit here than in the pedaling task. A higher cost should be expected in triple rather than in dual conditions because of the difficulty to increase and also in the advanced rather than in the early stages of PD. Group differences and dual or triple costs should be reduced or removed for the cognitive concurrent task and even for the hybrid cognitive-motor task when general cognitive status is statically controlled.

## 2. Methodology

### 2.1. Participants

Thirty-seven individuals (19 male and 18 female) with idiopathic PD were recruited for this baseline analysis. All patients with PD had been clinically diagnosed with idiopathic PD by a neurologist and were tested in the practical ON levodopa state, after ingesting antiparkinsonian medication (they reported that levodopa had taken its full effect). Subjects were recruited from Parkinson associations. They were invited to participate if they were between the ages of 50 and 85 years, were on levodopa treatment, experienced motor response fluctuations, and were able to ambulate independently. Five patients were excluded because they did not complete all the tasks.

Participants were classified as mild disability (stages I and II of the disease) and moderate disability (stages III and IV) according to the Hoehn and Yahr scale [22]. This scale is used internationally for the global assessment of PD and was designed to measure impairments associated with the disease progression. None of the participants presented severe disability (stage V).

All patients gave their written informed consent to participate in this study according to the Declaration of Helsinki. The protocol of this study was approved by the Research Ethics Committee of the Faculty of Education and Sport Sciences and then assigned code number 12-2205-17.

### 2.2. Assessment

#### 2.2.1. Gait

This variable was assessed using the Walk protocol with Wiva® sensors [34], a set of wireless inertial detection devices placed in the L4-L5 spinal segment. Wiva® sensors include an accelerometer, a magnetometer, and a gyroscope that allow professionals and practitioners to gather information about spatiotemporal parameters of the gait achieved during the Walk protocol. The Walk protocol consists of the patient moving in a straight line along a corridor of 80 cm for a distance of 10 m at a constant speed. This protocol is repeated twice, and the average of the obtained data is recorded. Variables recorded are stride speed (m/s), cadence (strides/min), stride length (m), simple support duration (s), double support duration (s), and gait cycle duration (s). All thes information was saved and sent to a PC via Bluetooth with Biomech Study 2011 v.1.1.

#### 2.2.2. Cognitive Status

The cognitive status of the participants was measured with the Montreal Cognitive Assessment (MoCA) [35]. The MoCA test is a widely used tool comprising 22 items and employed to screen patients with suspected mild cognitive impairment. The total score for the MoCA ranges from 0 to 30 points. It includes items from the following cognitive domains: memory, naming, language, visuospatial/executive functions, abstraction, attention/concentration/calculation, and orientation. In this study, we used Spanish normative scores for age and educational level [36].

#### 2.2.3. Motor Task

Tasks that mainly involve a mechanical or repetitive physical movement by the patient are representative of pure motor tasks. It applies, therefore, to any work that necessarily involves a movement of the body. Due to the characteristics of the patients, the selected motor task was pedaling, which consisted of indoor cycling [37, 38] for a period of 60 seconds and undertaken in the sitting position. The patient, seated in a chair with armrests, adjusts the pedals and performs a small warm-up before starting the test. The test consists of a series of cycles (without resistance) back and forth to become familiar with indoor cycling. Then, the cycle counter is set to “0” and the participant is told that he/she must pedal continuously for 60 seconds at a constant pace and at a cadence that allows the subject to speak at the same time that he/she is pedaling.

#### 2.2.4. Cognitive Task

The cognitive task chosen for the development of the study was a phonemic verbal fluency task. Verbal fluency tasks are often included in neuropsychological assessment to indicate cognitive impairment in persons with neurodegenerative diseases such as PD [39]. The task undertaken in this study was a task of phonemic fluency, in which each patient had to say as many words as possible over 60 seconds, having to start them by the assigned letters P, R, or M, having indicated previously that no proper names or words derived from others were valid (verbal conjugations and words from the same family). The frequency of Spanish words that begin with the letters P, R, and M is similar. To avoid the learning effect, each letter was used only in one of the experimental conditions.

#### 2.2.5. Motor-Cognitive Task

The task administered consists of using a pencil to draw a line through circles arranged in a path around a sheet of A3 paper containing 319 circles [33]. After a practice trial, the participants have 60 seconds to go through the circles along the path without lifting the pencil. The number of circles crossed with the pencil within the 60 seconds is calculated.

#### 2.2.6. Dual and Triple Tasks

The same measures were recorded in the phonemic fluency (number of correct words), tracking (number of circles crossed) and pedaling (number of pedal strokes) in the simple, dual and triple conditions. The phonemic fluency was measured by the number of correct words starting with the designated letter produced in 60 seconds. In the tracking task, the number of circles crossed with the pencil within the 60 seconds was recorded. In the pedaling task, the number of pedal strokes was recorded after the 60 seconds.

#### 2.2.7. Procedure

The tasks were performed by each patient individually, in a spacious and comfortable location and always supervised by the evaluators. The patients sat in a chair without armrests with a height of 47 cm and with the backrest fixed to the wall. In front of the patients was a desk 72 cm high. Tasks were administered to the participants seven days a week and always in the morning. The protocol was carried out within two weeks, and all measurements for each patient were taken on the same day.

All the tasks and conditions were applicable to the sample participants [40]. Tasks were performed first in the single condition and in the following order: (1) phonemic fluency, (2) tracking, and (3) pedaling. Subsequently, the tasks were performed in the dual mode in a counterbalanced manner, combining them as follows: phonemic fluency + tracking and phonemic fluency + pedaling or phonemic fluency + pedaling and phonemic fluency + tracking. Finally, for the triple-task condition, participants were asked to perform the phonemic fluency, the tracking, and the pedaling tasks simultaneously. In these dual and triple conditions, participants were asked to perform the phonemic fluency jointly with the another concurrent task (either with tracking or with pedaling task) for 60 s.

#### 2.2.8. Statistical Analysis

A descriptive analysis was carried out through measurements of central tendency (mean) and dispersion (standard deviation) to describe the sample, thus stratifying the description according to the stage in which the sample is located. Student's *t*-tests were conducted by comparing mild and moderate disability groups for age (years), years of education, MoCA score, and gait: spatiotemporal parameters, phonemic fluency (simple, dual with tracking, dual with pedaling, and triple), tracking (simple, dual, and triple), and pedaling (simple, dual, and triple). Mixed ANOVA analyses were performed to test group differences (mild and moderate disability) between single and dual conditions for phonemic fluency, tracking, and pedaling tasks in the following combinations: phonemic fluency-tracking and phonemic fluency-pedaling, considering the MoCA total score as the covariate. Similarly, mixed ANOVA analyses were also performed to test group differences between single and triple conditions for each of the following tasks: phonemic fluency, tracking, and pedaling, also controlling for MoCA total score. The estimated marginal means represented in the figures correspond to the average scores for each level of combination of the factors considered (i.e., disability and condition), adjusted for the covariate of the cognitive status. Multiple-comparison post hoc Bonferroni correction was used to evaluate pairwise significant comparisons. The alpha value was established at .05 for all analyses, and the partial eta squared value (*η*_*p*_^2^) is reported as an estimate of effect size in the mixed ANCOVAs.

## 3. Results

### 3.1. Group Comparisons

Comparisons between groups with mild and moderate disability are shown in Table 1. Although, in general, performances were better in the mild disability group, significant differences were only reached in stride speed, tracking in the single condition, and pedaling in both the single and the triple conditions.

### 3.2. Comparisons between Single and Dual Conditions

**Figure 1:**
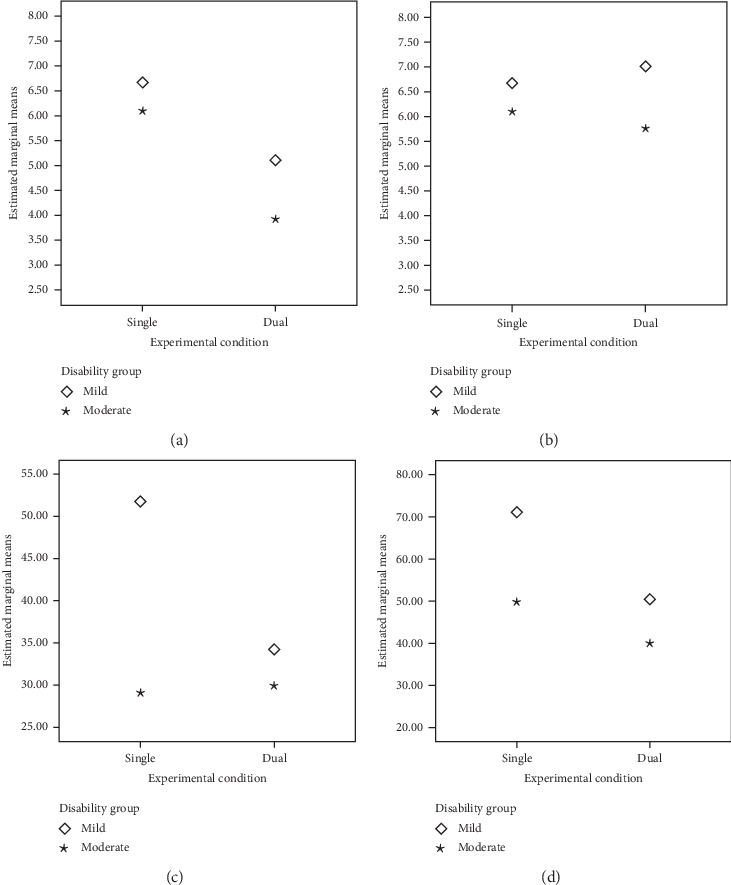
Estimated marginal means (mixed ANCOVA) for concurrent tasks in single and dual conditions by the disability group. (a) Phonemic fluency (with tracking). (b) Phonemic fluency (with pedalling). (c) Tracking (with phonemic fluency). (d) Pedaling (with phonemic fluency).

Mixed ANOVA analyses comparing group differences between single and dual conditions (see Table 2 and Figure 1) revealed (a) main effects for the disability group factor were found only in tracking and pedaling, but not in fluency despite being combined with tracking or pedaling; (b) significant differences in fluency between single and dual conditions when combined with tracking, but no significant group interactions; (c) no significant differences were found for phonemic fluency when combined with pedaling; (d) significant differences were observed between single and dual condition in the tracking task when combined with fluency, and in the interaction between condition and disability group; and (e) significant differences in the pedaling task when combined with fluency only for condition, but no significant group interactions.

The significant differences in condition disappeared in the mixed ANCOVA when the MoCA score was included as covariate, whereas the main effects for the disability group factor in tracking, and pedaling tasks, and the significant interaction between the task condition and the disability group for tracking, remained significant (see Table 2). Post hoc Bonferroni tests showed (a) better performance in the mild disability group only in the single condition and (b) differences between single and dual conditions only in the mild disability group.

### 3.3. Comparisons between Single and Triple Conditions

**Figure 2:**
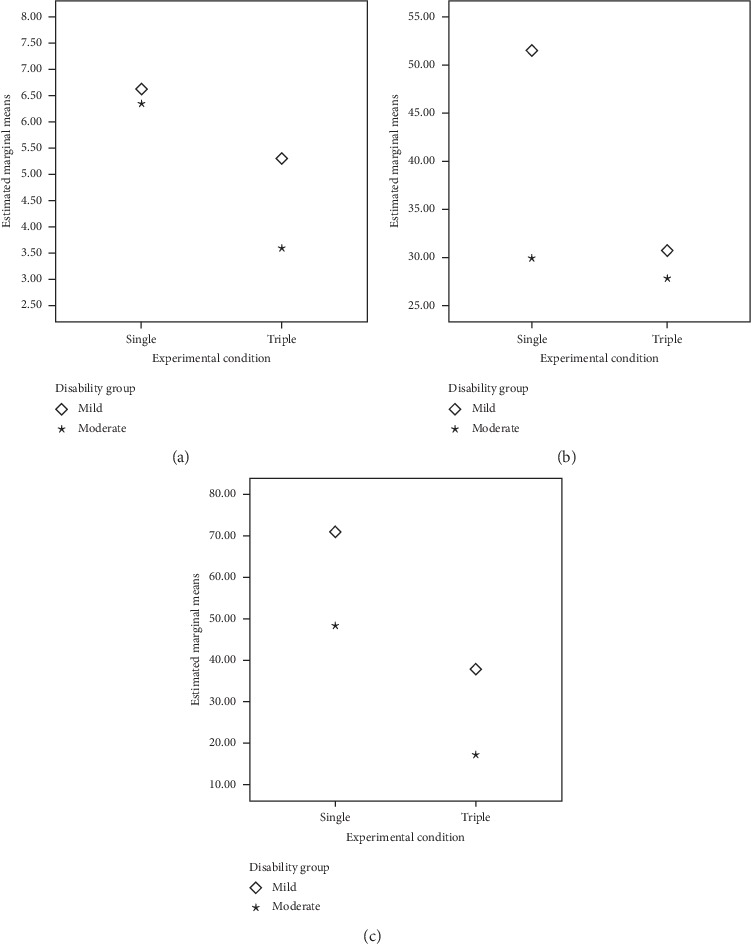
Estimated marginal means (mixed ANCOVA) for concurrent tasks in single and triple conditions by the disability group. (a) Phonemic fluency (with pedaling and tracking). (b) Tracking (with phonemic fluency and pedaling. (c) Phonemic with phonemic fluency tracking.

Similar to those results described above for dual conditions, comparisons between single and triple conditions revealed that (see Table 3 and Figure 2): (a) the main effect for the disability group factor was found only in pedaling, but not in fluency or tracking; (b) significant differences between single and triple conditions for phonemic fluency; (c) significant differences in the tracking task both for condition and for the interaction between condition and group factors; and (d) significant differences between single and triple conditions for the pedaling task.

Similar to those results found for the comparisons between dual and simple conditions, the significant differences in experimental conditions disappeared in mixed ANCOVA when the MoCA score was included as a covariate (see Table 3). Only the condition-group interaction remained significant for tracking. Bonferroni's pairwise comparisons showed (a) better performance in the mild disability group only in the single condition and (b) differences between single and triple conditions only in the mild disability group.

## 4. Discussion

Our results showed significantly worse performance in the more disabled PD group for the pure motor (pedaling) and hybrid (tracking) tasks regardless the experimental condition (i.e., single or dual). However, disabled groups showed similar performance in the cognitive task (fluency), regardless of the experimental condition. In line with previous studies [23, 24], our results pointed out that the measurement of phonemic fluency seems to be relatively unimpaired in PD and is not sensitive enough to be able to differentiate between the mild and moderate disability stages of the disease in a dual setting context.

Similar dual costs (i.e., worsening of performance in dual compared to single condition) in both disability groups were observed in fluency (when combined with tracking), pedaling (when combined with fluency), and tracking tasks (when combined with fluency), but not for the fluency task when combined with pedaling. As expected [11], the combination of the fluency task with pedaling, a pure motor task largely preserved in PD patients [29, 30], seems to be responsible for the similarity in fluency performance regardless of the experimental condition. In addition, all performance differences by condition for fluency when combined with tracking, pedaling when combined with fluency, and tracking when combined with fluency were neutralized when the cognitive status measure was included in the model as a covariate, supporting the suggestion that dual costs in PD could be largely related to cognitive status even for those associated to the almost pure motor task, namely, the pedaling task [7–9].

In line with what it was hypothesized, the tracking measure when performed with the fluency task was the only combination sensitive to the differences in condition between the disability PD groups. Unlike what was observed in pedaling when combined with fluency, the tracking task was sensitive to detect significantly better performance in the mild than in the moderate disability group for the single condition and to show a significant decrease in performance in the dual condition for the mild disability group that reaches levels similar to those shown by the moderate disability group. Thus, the tracking task was the only one able to detect differences between PD disability groups in concurrent-task paradigms.

On the other hand, motor (i.e., pedaling) and, particularly, cognitive (fluency) task failed in discrimination between disability groups in multitask setting. Thus, although pedaling performance was significantly poor in mild than in moderate disability group even when cognitive status was statistically controlled, differences were similar in single and dual conditions. Regarding fluency task, disability group differences were neutralized when cognitive status covariates were considered.

Only the hybrid task demonstrated the ability to differentiate between disability groups in multitask setting. We suggest that the subtle interplay between difficulty, novelty, and cognitive-motor demands should be critical in determining the sensibility to the motor, cognitive, and interaction of cognitive-motor impairments in PD.

The interaction between group and condition in the tracking task was robust and remained significant when the cognitive status was statistically controlled, suggesting that its specific motor-cognitive hybrid nature is responsible for capturing the differences between disability groups by condition. However, contrary to what has been reported in healthy older subjects [12] and to the expectations regarding the importance of the difficulty dimension in the taxonomy by McIsaac et al. [11], results remained almost the same in the triple task condition, suggesting that the increase of difficulty associated with the triple condition does not have any influence on the disability group comparison. It can be expected that, beyond the motor or cognitive nature of the concurrent tasks, other task specifications would be useful to overcome the lack of consensus on their ability to detect deficiencies associated with PD [14, 16, 17]. Our results suggest that the difficulty and novelty dimensions could be partially responsible for the detection of differences between PD disability groups in a dual paradigm. However, since the main results remained unchanged for almost all task combinations in the trial condition, it is thought that dimensions such as difficulty do not have such a relevant role in the modulation of the dual effect.

Our results highlight the importance of jointly measuring the complex interplay between motor and cognitive skills in PD [4, 5], not only in multitask settings but also in single condition performance. Only tasks that impose motor-cognitive hybrid demands seem to be able to detect disability levels in single condition and performance worsening in concurrent condition (i. e., dual or triple), even when cognitive decline is controlled. Tasks with a purer cognitive or motor component appear to be inadequate to detect the changes associated with PD progression in multitask settings.

Some limitations of this study should be overcome in the future. First, the sample size must be enlarged and control of physical activity or specific motoric interventions should be considered because it could affect differently the progression of motor symptoms in each of the disability groups. Likewise, the inclusion of a healthy control group is considered of interest in order to study dual performance differences with PD patients in concurrent tasks. To add the motor-hybrid task pairing would be useful in order to have a complete picture of the motor-cognitive task combination possibilities. Finally, the substitution of the phonemic fluency task for the action fluency modality should be relevant because it has shown higher sensibility to progression in PD.

## 5. Additional Points

(1) Hybrid motor-cognitive task differentiated between mild and moderate PD patients. (2) Mild group performance worsened in dual condition to match that of the moderate group. (3) Between-group differences in the hybrid task were not neutralized by cognitive status

## Figures and Tables

**Table 1:** Comparisons between mild and moderate disability groups, including means, standard deviations between brackets, and *t* scores for Student *t*-tests.

	Mild disability	Moderate disability	*t*
Age (years)	69.42 (10.64)	71.00 (7.38)	−0.46
Years of education	9.36 (2.43)	11.15 (4.24)	−1.51
MoCA score	18.90 (7.53)	17.23 (8.52)	0.58
Gait (spatiotemporal parameters)			
Stride speed (m/s)	0.83 (0.28)	0.63 (0.22)	2.19^*∗*^
Cadence (strides/min)	91.56 (11.27)	78.34 (14.71)	1.44
Stride length (m)	1.09 (0.23)	1.04 (0.22)	0.61
Simple support duration (s)	0.75 (0.09)	0.71 (0.08)	0.18
Double support duration (s)	0.23 (0.08)	0.24 (0.07)	−0.87
Gait cycle duration (s)	1.26 (0.16)	1.49 (0.42)	−0.90
Phonemic fluency			
Single	6.89 (4.20)	5.77 (4.71)	0.71
Dual (with tracking)	5.26 (4.23)	3.69 (3.59)	1.10
Dual (with pedaling)	7.21 (5.01)	5.46 (4.20)	1.03
Triple	5.42 (3.66)	3.41 (3.68)	1.48
Tracking			
Single	53.05 (29.77)	27.23 (17.32)	2.81^*∗∗*^
Dual	35.74 (26.46)	27.69 (18.93)	0.94
Triple	32.42 (24.33)	25.17 (20.29)	0.86
Pedaling			
Single	71.95 (24.86)	48.62 (20.65)	2.79^*∗∗*^
Dual	51.05 (16.70)	39.08 (19.34)	1.87
Triple	38.10 (17.97)	16.75 (14.67)	3.45^*∗∗*^

^*∗*^ *p* < 0.05; ^*∗∗*^*p* < 0.01.

**Table 2:** Mixed ANOVAs and ANCOVAs comparing single and dual condition (intrasubject factor) for the fluency-pedaling and fluency-tracking dual tasks considering the disability groups (intersubject factor).

Tasks	Mixed ANOVAs	Mixed ANCOVAs^*∗*^
Fluency (with pedaling)	—	—
Fluency (with tracking)	Condition: single > dual	—
	[*F* (1, 30) = 7.94, *p*=0.008, *η*_*p*_^2^ = 0.20, observed power = 0.78]	
Pedaling (with fluency)	Disability group: mild > moderate	Disability group: mild > moderate
	[*F*(1, 29) = 6.86, *p*=0.014, *ηp*^2^ = 0.18, observed power = 0.71]	[F(1, 29) = 6.96, *p*=0.013, *η*_*p*_^2^ = 0.19, observed power = 0.72]
	Condition: single > dual	—
	[*F*(1, 30) = 21.36, *p* < 0.001,*η*_*p*_^2^ = 0.42, observed power = 0.99]	
Tracking (with fluency)	Disability group: mild > moderate	
	[*F*(1, 29) = 5.19, *p*=0.030, *η*_*p*_^2^ = 0.15, observed power = 0.59]	
	Condition: single > dual	Condition: single > dual
	[*F*(1, 30) = 6.90, *p*=0.013, *η*_*p*_^2^ = 0.19, observed power = 0.72]	[*F*(1, 29) = 4.19, *p*=0.049, *η*_*p*_^2^ = 0.12, observed power = 0.50]
	Disability group *x* condition:	Disability group *x* condition:
	[*F*(1, 30) = 7.68, *p*=0.010, *η*_*p*_^2^ = 0.20, observed power = 0.77]	[*F*(1, 29) = 8.02, *p*=0.008, *η*_*p*_^2^ = 0.22, observed power = 0.78]
		Bonferroni:
		(a) Low > high in single condition [*F*(1, 29) = 8.90, *p*=0.006, *η*_*p*_^2^ = 0.23, observed power = 0.82]
		(b) Single > dual in the mild group [*F*(1, 29) = 18.16, *p* < 0.001, *η*_*p*_^2^ = 0.38, observed power = 0.98]

^*∗*^MoCa total score as the covariate.

**Table 3:** Mixed ANOVAs and ANCOVAs comparing single and triple condition (intrasubject factor) for the fluency-pedaling-tracking triple task considering the disability groups (intersubject factor).

Tasks	Mixed ANOVAs	Mixed ANCOVAs^*∗*^
Fluency (with pedaling and tracking)	Condition: single > triple	—
	[*F*(1, 29) = 7.52, *p*=0.01, *η*_*p*_^2^ = 0.21, observed power = 0.76]	
Pedaling (with fluency and tracking)	Disability group: mild > moderate	Disability group: mild > moderate
	[*F*(1, 29) = 14.03, *p* < 0.001, *η*_*p*_^2^ = 0.33, observed power = 0.95]	[*F*(1, 28) = 13.39, *p* < 0.001, *η*_*p*_^2^ = 0.32, observed power = 0.94]
	Condition: single > triple	—
	[*F*(1, 29) = 57.95, *p* < 0.001, *η*_*p*_^2^ = 0.66, observed power = 1.0]	
Tracking (with fluency and pedaling)	Condition: single > triple	—
	[*F*(1, 29) = 12.38, *p* < 0.001, *η*_*p*_^2^ = 0.30, observed power = 0.93]	
	Disability group *x* condition:	Disability group *x* condition:
	[*F*(1, 29) = 7.86, *p*=0.009, *η*_*p*_^2^ = 0.21, observed power = 0.77]	[*F*(1, 28) = 7.86, *p*=0.009, *η*_*p*_^2^ = 0.22, observed power = 0.77]
		Bonferroni:
		(a) Low > high in single condition [*F*(1, 28) = 25.33, *p* < 0.001, *η*_*p*_^2^ = 0.47, observed power = 0.99]
		(b) Single > dual in the mild group [*F*(1, 28) = 7.48, *p*=0.009, *η*_*p*_^2^ = 0.21, observed power = 0.75]

^*∗*^MoCa total score as the covariate.

## Data Availability

The PHONEMIC, FLUENCY, TRACKING, AND PEDALING data used to support the findings of this study are restricted by the RESEARCH ETHICS COMMITTEE OF THE FACULTY OF EDUCATION AND SPORT SCIENCES in order to protect PATIENT PRIVACY. Data are available from the HealthyFit Group, e-mail: ghi22@uvigo.es, for researchers who meet the criteria for access to confidential data.
